# Driving ban for diesel-powered vehicles in major cities: an appropriate penalty for exceeding the limit value for nitrogen dioxide?

**DOI:** 10.1007/s00420-018-1297-4

**Published:** 2018-02-22

**Authors:** Matthias Möhner

**Affiliations:** Division of Work and Health, Federal Institute for Occupational Safety and Health, Nöldnerstr, 40/42, 10317 Berlin, Germany

To protect the general population from adverse health effects, Germany set a limit value of 40 μg/m³ for the annual mean exposure to nitrogen dioxide (NO_2_) based on WHO’s air-quality guidelines and in accordance with the European directives (DE 2010; EC 2008; WHO 2006).

According to the German Environment Agency, in 2017 this limit value was exceeded at 46% of the urban traffic-related air monitoring stations (Minkos et al. 2018). The European Directive recommends severe sanctions for exceeding the limit value: “The penalties provided for must be effective, proportionate and dissuasive” (EC 2008). Since diesel-powered vehicles are regarded as the main traffic-related source of nitrogen oxides, driving bans for inner-city areas in many large German cities such as Hamburg, Munich, and Stuttgart are the subject of intense debate. In the passenger car sector alone, 80% of the currently around 15 million diesel-powered passenger cars registered are unlikely to meet the EURO 6 standard (Kraftfahrt-Bundesamt 2017) and would, therefore, be threatened by a local driving ban.

In contrast to Europe, the corresponding limit value for NO_2_ in the United States has been 100 μg/m³ since the 1970s. For the forthcoming periodic review of limit values, the US Environmental Protection Agency (EPA) recommends retaining the current value for NO_2_ (EPA 2016, 2017). Even the highest annual average value measured in Germany in 2016 [Stuttgart, Neckartor: 82 μg/m³ (Minkos et al. 2017)] was below the US-value, i.e. if the US value were also valid in Europe, a ban for diesel-powered vehicles would not be an issue in Germany at all. If the difference in limit values can have such serious consequences, it is indeed necessary to question why the WHO and EPA boards arrived at different conclusions, even though the body of available studies is approximately equal. The difference in the two assessments is most likely due to differences in the critical examination of risk of bias in epidemiological studies. There are no epidemiological studies that can be confidently used quantitatively to estimate long-term NO_2_ exposure durations or concentrations likely to be associated with the induction of unacceptable health risks in children or adults. The most relevant publications in this context, which argue for adverse health effects of NO_2_, are based on the Harvard Six Cities Study. The investigators analyzed combined respiratory symptoms of children aged 7–11 years based solely on parental reports with incompletely assessed exposure to gas stove emissions (Neas et al. 1991). These results form the basis for WHO’s first recommendation of 40 μg/m³ as a long-term limit value for NO_2_ (WHO 1997). Later, these study results gained further support by the application of measurement error correction methods to the study data (Li et al. 2006). In contrast, a large prospective study in infants exposed to a similar environment showed no health effects related to NO_2_ exposure (Samet et al. 1993). However, the WHO group argued that the likelihood of causation of adverse health effects by NO_2_ is strengthened because (a) epidemiological results are replicated across different studies with variable underlying conditions; (b) multiple health outcomes appear to be affected in a consistent and coherent manner; and (c) the results are supported by either toxicological or controlled studies on humans (WHO 2006). In the EPA report, the complex problems involved in risk assessment are discussed very decidedly: there is a need to consider (a) exposure factors potentially correlated with NO_2_ exposure in evaluating relationships with health effects, (b) factors contributing to error in estimating exposure to ambient NO_2_ and (c) confounding bias (EPA 2016).

NO_2_ is regarded as a marker for air pollution caused by motorized road traffic. The combustion processes also release particulate matter (PM) with aerodynamic diameter < 10 μm (PM_10_) or the even smaller part (PM_2.5_) as well as the gaseous pollutants sulfur dioxide (SO_2_), nitrogen monoxide (NO), ozone, carbon monoxide (CO), and volatile organic compounds (VOCs). The correlation between these components at the monitoring points is typically greater than 0.80. Moreover, road abrasion, brake wear and tire wear are non-exhaust traffic emissions that cause additional exposure to particulate matter containing silica, iron, zinc, copper, elemental carbon, barium and others. Toxicological and epidemiological research increasingly indicates that such non-exhaust pollutants could be responsible for some of the observed adverse effects on health (Gent et al. 2009; WHO 2013). It should be noted that for some health impairments, traffic noise must also be considered as a potential cause. In its conclusion the EPA, therefore, emphasizes that the extent to which NO_2_ may be serving primarily as a surrogate for the broader traffic-related pollutant mix remains unclear (EPA 2017).

The measured values for NO_2_ as well as for other traffic-related pollutants are usually gathered via the permanently installed monitoring network, which primarily takes into account heavily exposed road sections. In terms of their spatial distribution, however, the pollutants differ considerably from one another. The exposure to PM_10_ is fairly homogeneous, i.e. a measured value at a central site is representative of a larger area. But NO_2_ spreads close to the ground locally. The variation of the exposure in inner-city areas depends not only on the distance from a major road, traffic characteristics, industrial point sources and housing or population density and some other land-use data available from geographic information systems (GIS), but also very much on the concrete development and design of the green spaces at micro level. For example, a study in Bavaria has shown that in the inner courtyard of a house, for which very high values were measured on the street side with above 80 μg/m³ NO_2_, the measurements were at the level of the urban background (Schädel et al. 2015). Thus, central site monitors and improved estimates derived from land-use regression models may not represent the magnitude of long-term average NO_2_ concentrations for study subjects in their residential environment. Consequently, the observed correlation between NO_2_ and other traffic-related pollutants at central cites may not represent the real correlation for study subjects.

In addition to the impact of other traffic-related pollutants, the socio-economic status (SES) of study subjects must also be taken into account when comparing different urban districts. Results of studies conducted in North America and Europe show a negative correlation between NO_2_ exposure and SES in terms of education, employment class or household income (Finkelstein et al. 2005; Naess et al. 2007). It has also been shown that SES is inversely correlated with morbidity and mortality of diseases such as cancer or cardiovascular diseases, mainly due to lifestyle factors (Galobardes et al. 2006; Power et al. 2005; Vohra et al. 2016). One such confounding factor to be taken into consideration is smoking. Unfortunately, most studies provide, if at all, very rough information on smoking (current smoker, ex-smoker, never smoker). Hence, residual confounding is an issue for many studies.

With regard to the risk-of-bias discussion carried out in the WHO report (WHO 2006), it should be noted that the effect direction of confounding variables does not differ between studies and that comparable methods of exposure assessment have been used, so that the bias directions are likely to be similar. Moreover, smoking and SES are known risk factors for most diseases for which correlation with NO_2_ exposure has been observed.

When discussing the health effects of NO_2_, it is also important to bear in mind the impact of carbon dioxide (CO_2_) emissions on the environment. Diesel is burned more efficiently in the engines than gasoline, which is why the diesel engine emits less CO_2_ than a comparable gasoline engine. The increasingly efficient engines have led to a reduction of the specific emission (emission per passenger-kilometer) of CO_2_ since 1995 of 13 percent for passenger cars (UBA 2017). Other specific emissions were reduced even more significantly in this period, including NO_2_ at 60% and PM at 72%. However, the trend towards ever larger and more powerful passenger cars unfortunately implicates that the continuous increase in the efficiency of combustion engines could not be translated into an overall reduction in fuel consumption. During the same period, the annual mean concentration in the atmosphere of global CO_2_ rose by more than 13% (NOAA 2017) contributing to climate change with observed and projected adverse impacts on health. If the concern is human health, the primary goal must, therefore, be to reduce global CO_2_ emissions noticeably. In terms of road traffic as one of the main sources of these emissions, this means that a turnaround towards lighter and, therefore, less motorized vehicles must be initiated. The combustion engines must be made more efficient and their emissions must be further reduced. A general speed limit on motorways could help to slow down the drive for ever more powerful engines in passenger cars. In addition, the attractiveness of public transport must also be significantly increased. However, the biggest challenge that needs to be addressed is the rapid development of truly climate-neutral mobility, including the long-term and environmentally compatible extraction of the raw materials required as well as efficient energy storage. More research is needed in this field. Research initiatives such as Sodium–Magnesium Hybrid Batteries (Walter et al. 2015) or synthetic, CO_2_-neutral fuel for combustion engines (Leitner et al. 2017; Pischinger 2016) are promising approaches. Like the diesel engine, e-mobility, which is based on lithium-ion batteries, will probably only prove to be a bridging technology. The most important raw materials, especially lithium and cobalt, are probably not available in sufficient quantities and the way in which they are extracted is partly subject to problematic conditions.

In view of these challenges to the sustainability of our mobility and taking into account the described weaknesses of epidemiological evidence for an adverse health effect of NO_2_ emissions, a ban on the use of diesel-powered vehicles seems disproportionate.
